# Speech Evoked Auditory Brainstem Response in Stuttering

**DOI:** 10.1155/2014/328646

**Published:** 2014-08-19

**Authors:** Ali Akbar Tahaei, Hassan Ashayeri, Akram Pourbakht, Mohammad Kamali

**Affiliations:** ^1^Department of Audiology, School of Rehabilitation Sciences, Iran University of Medical Sciences, Tehran 15459-13187, Iran; ^2^Department of Basic Sciences in Rehabilitation, School of Rehabilitation Sciences, Iran University of Medical Sciences, Tehran 15459-13187, Iran; ^3^Rehabilitation Research Center, School of Rehabilitation Sciences, Iran University of Medical Sciences, Tehran 15459-13187, Iran

## Abstract

Auditory processing deficits have been hypothesized as an underlying mechanism for stuttering. Previous studies have demonstrated abnormal responses in subjects with persistent developmental stuttering (PDS) at the higher level of the central auditory system using speech stimuli. Recently, the potential usefulness of speech evoked auditory brainstem responses in central auditory processing disorders has been emphasized. The current study used the speech evoked ABR to investigate the hypothesis that subjects with PDS have specific auditory perceptual dysfunction. *Objectives*. To determine whether brainstem responses to speech stimuli differ between PDS subjects and normal fluent speakers. *Methods*. Twenty-five subjects with PDS participated in this study. The speech-ABRs were elicited by the 5-formant synthesized syllable/da/, with duration of 40 ms. *Results*. There were significant group differences for the onset and offset transient peaks. Subjects with PDS had longer latencies for the onset and offset peaks relative to the control group. *Conclusions*. Subjects with PDS showed a deficient neural timing in the early stages of the auditory pathway consistent with temporal processing deficits and their abnormal timing may underlie to their disfluency.

## 1. Introduction

Developmental stuttering is a subtype of speech fluency disorders characterized clinically by a disruption in the verbal fluency. Despite a great volume of research and various theories, the underlying mechanisms for stuttering are still not fully understood. Although prior to adolescence and with increasing age, the disorder remediates in the majority of the children; there is a subset of stuttering children who do not recover and their stuttering behaviours persist into adulthood. Regardless of gender and handedness, approximately the prevalence of persistent stuttering in adults has been estimated to be about 1% [1].

Recent advances in neurological studies have shown abnormal neural activity in the auditory and motor areas as well as subcortical regions such as the basal ganglia in developmental stuttering [2, 3]. Neuroimaging and magnetoencephalography (MEG) studies have demonstrated that subjects with persistent developmental stuttering (PDS) show functional and structural peculiarities in the central nervous system including unusual activation patterns in the auditory and motor areas as well as gyral and planum temporale anomalies [4–7].

These findings were essentially replicated in several subsequent studies. At least, two imaging studies point to structural brain anomalies in PDS subjects. In the first study, Foundas et al. found that PDS is associated with anomalous planum temporale (PT) asymmetry [2]. The PT seems to play an important role in the representation of speech information at the higher levels of auditory processing and is thought to be important in coordinating incoming auditory feedback with speech output. In another study, using a novel imaging* method named* diffusion tensor imaging (DTI), Sommer et al. found that the white matter tracts in the left operculum in stutterers were less dense than those in controls [8].

Several electrophysiological studies have shown cortical dysfunctions in stuttering. Recent studies have reported mismatch negativity (MMN) abnormalities in PDS subjects that are more prominent in the left hemisphere. MMN, another negative component of ERPs, is believed to index automatic process involved in verbal sensory memory. Using simple pure tone stimuli and speech stimuli, Corbera et al. compared the MMN response between subjects with PDS and fluent speakers [9]. Although they found normal MMN responses for tonal stimuli in both groups, PDS subjects showed abnormal left mastoid MMN amplitude for speech stimuli. This finding is consistent with abnormalities primary auditory cortex of the left temporal-parietal region, a region involved in verbal sensory memory. Cuadrado and Weber-Fox investigated p600 late language related wave in subjects who stutter [10]. They found smaller p600 amplitudes in stutterers, suggesting atypical syntactic processing. Morgan et al. recorded P300 potentials from the left and right hemisphere in 8 stutters and compared the results with 8 matched controls [11]. They found larger p300 amplitudes in 60% of stutters (5 out of 8) in the left hemisphere. In contrast to stutterers in all normal fluent speakers the amplitude of P300 was larger in the right hemisphere.

Stuttering has also been considered as a timing deficit [12, 13]. Mistiming in stuttering is not restricted to the speech motor areas. Evidence in favor of timing deficits in the auditory cortex was reported by Beal et al. in a MEG study [14]. They measured auditory evoked magnetic fields in PDS subjects and fluent speakers during passive listening and active speaking tasks. Their results showed that adults with PDS had slower cortical timing (longer auditory M100 latencies) compared to fluent speakers, indicating impaired auditory motor integration. Also, according to the suggestion by Kent, subjects who stutter may be poorer at auditory temporal processing [15]. In addition, the result obtained from studies of central auditory processing in stuttering showed that stuttering adults differed from adults who not stutter in some aspects of auditory temporal information processing [9, 16–18].

Several investigators pointed to the auditory brainstem as a possible origin for a central auditory deficit in stutterers [16, 19, 20]. Electrophysiological tests such as auditory brainstem response (ABR) and frequency following response (FFR) have shown to be highly efficient in identifying brainstem timing deficits. A number of papers have offered convincing evidence that shows that the measurement of speech-ABR is a reliable technique for assessing brainstem timing in clinical populations who are suspected of auditory processing impairments [21]. The speech evoked ABR measurements offer a quantitative evaluation of the auditory pathways at the rostral part of the brainstem and are probably the most reliable of brainstem timing measures at this level. Apart from brainstem timing measures the speech-ABR offers invaluable information about pitch and harmonic encoding.

Three studies using synthetic sentence identification-ipsilateral competing message (SSI-ICM) observed that stutterers perform worse than nonstutterers [22–24]. In another study, the binaural masking level difference (BMLD) test at 500 Hz was administered to 10 adults with developmental stuttering [16]. Adults who stutter displayed smaller MLDs relative to controls. This discrepancy suggests that PDS subjects may have a deficit in binaural interaction processing. The BMLD is the improvement in the discrimination of a signal in the presence of the noise under dichotic listening conditions when the noise or signal deliver out of phase [25]. The encoding of interaural phase difference (ITD) as reflected by BMLD requires temporal processing in the order of microsecond and depends upon the integrity of ITD-sensitive neurons in the brainstem nuclei [26]. Both tests are sensitive to brainstem dysfunction and need the subjects to use temporal information. These findings provide further support for an involvement of the brainstem in stuttering. The current study used the speech-ABR to test the hypothesis that subjects with PDS have specific auditory perceptual dysfunction at the level of brainstem.

## 2. Methods and Materials

### 2.1. Subjects

In this cross-sectional study, twenty-five individuals with PDS (21 male and 4 female; mean age: 24.48 ± 3.99 years old; age range: 16–35 years) were recruited from the IRAN Society of Stuttering and the Speech Therapy Department of Iran University of Medical Sciences. Twenty-five fluent subjects (21 male and 4 female; mean age: 22.44 ± 2.32 years old; age range: 16–35 years) served as the control group (Table 2). All volunteers used Persian as a native language and had normal hearing sensitivity (pure tone thresholds) at octave intervals ranging from 250 to 4000 Hz (≤20 dB HL) and were free from otological or neurological problems. Both groups matched for education and sex distribution. All subjects were right-handed as checked by the Persian version of Edinburgh handedness questionnaire [27]. The clinical diagnosis of developmental stuttering and the severity of stuttering were assessed by speech language pathologists. Stutterers suffer from disease for more than ten years and none of them had intensive treatment programs for at least last year. Stuttering severity was measured by Persian version of the Stuttering Severity Instrument-3 (SSI-3) and ranged from mild to severe. All stuttering adults met the clinical criteria of developmental stuttering such as word-initial stuttering, presence of anxiety during stuttering, secondary behaviors, adaptation effect, and situational variations and were differentiated from neurogenic stuttering. Subjects with abnormal response to the click-ABR were excluded from the study. All stutterers and control subjects signed informed consent. The study was approved by the Medical Ethics Committee of Iran University of Medical Sciences.

### 2.2. Stimulus and Recording Parameters

The ABR data were gathered in a suitable room with a low background noise while subjects were watching a videotape. At the time of testing, subjects were positioned in a comfortable chair and were instructed to be motionless. Prior to the speech evoked ABR assessment, responses to the click-ABR were collected for all subjects. For the click evoked ABR two blocks of 2000 sweeps were delivered at 80 dB nHL to the right and left ears via insert ear phones (ER-3) at a rate of 13.3/s with alternating polarity and processed over a 10.66 ms averaging epoch. The recordings were collected using a band pass filter set to 100–3000 Hz. The speech-ABRs were obtained by the Bio-logic Navigator Pro AEP System (Version 7) with vertical electrode array of three surface Ag-AgCl electrodes (active: Cz; reference: ipsilateral earlobe; ground: forehead) using a band pass filter set to 100–2000 Hz and were digitally sampled at 12 KHz (1024 points over 85.33 ms). The artifact rejection was set online to ±25 *μ*V and all impedance electrodes were maintained under 3 KOhms. Two blocks of 3000 sweeps were delivered monaurally at 80 dB SPL to the right ear via insert ear phones (ER-3, Etymotic Research, Elk Grove Village, IL, USA) at a rate of 10.9/s with alternating polarity and averaged over an 85.33 ms time window (−15 ms pre-stimulus). The BioMARK responses were elicited by a 5 formant synthesized syllable/da/, with duration of 40 ms, which was produced using a Klatt synthesizer at the rate of 10 kHz (the default stimulus in the Biological system software). The fundamental frequency (F0), the first formant (F1), the second formant (F2), and the third formant (F3) shift linearly through the duration of the speech stimulus: the F0 and F1 rise from 105–125 Hz and 455–720 Hz, respectively, while the F2 decreases from 1700 to 1222 Hz. The F3 reduces from 2550 to 2000 Hz. The last two formants (F4 and F5)* remain* fixed at 3600 Hz and 4600 Hz.

### 2.3. Data Analysis

After recording BioMARK responses, all data were converted to a text file using AEP to ASCII (version 1.6.0) and then were transferred to the Brainstem toolbox (Skoe & Kraus, 2010) for further analysis. This toolbox is a MATLAB-based package for conducting temporal and spectral analyses. Using the brainstem toolbox we determined timing (peaks: V-A-D-E-F-O), composite (VA inter-peak latency, VA inter-peak amplitude, VA slope) and spectral (F0-F1-HF) measures. Due to low detectability of the peak C (present in 80% of subjects), this wave was excluded from statistical analysis.

To achieve a higher confidence in the timing measures, we objectively detected all peaks via automated peak-picking algorithms in the brainstem toolbox. To obtain more detailed information regarding the frequency encoding in the sustained segment of the response, fast Fourier transform (FFT) was employed and the spectral magnitudes of the FFR were measured over a 11.4–40.6 ms time window in the three ranges of frequencies (F0: 103–121 Hz, F1: 454–719 Hz, and HF: 721–1155 Hz). Due to the upper limit of phase locking (1500 Hz) at the level of the rostral brainstem, F2 and the higher formants, which have higher frequencies, were not measured.

The data from two groups were found to be normally distributed as assessed by a Kolmogorov-Smirnov test. So for statistical analysis parametric tests were used. Independent sample* t*-tests were applied to determine any significant differences in the timing, composite, and spectral measures between stutterers and nonstutterers for each of the test variables. A Pearson product-moment correlation was performed to assess the relationship between severity of stuttering as scores indexed in SSI-3 and brainstem timing measures. Data collected by this research were statistically analyzed via SPSS version 17. For all statistical tests, the significance level was set at *P* < 0.05.

## 3. Results

**Figure 1:**
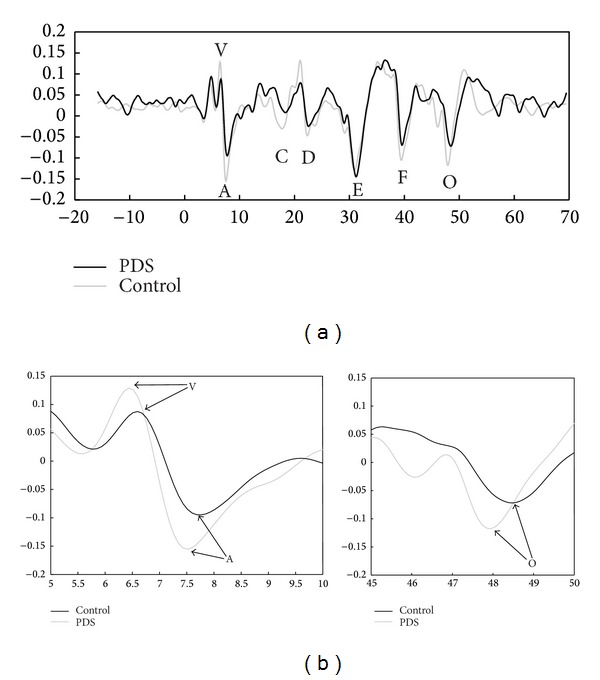
(a) Comparison of brainstem response to speech in controls (gray line) and subjects with persistent developmental stuttering (black line). (b) Auditory brainstem response to speech sounds in stutterers showed significant prolongations in the onset responses (waves V and A) as well as for the offset response (wave O). PDS = persistent developmental stuttering.

**Figure 2:**
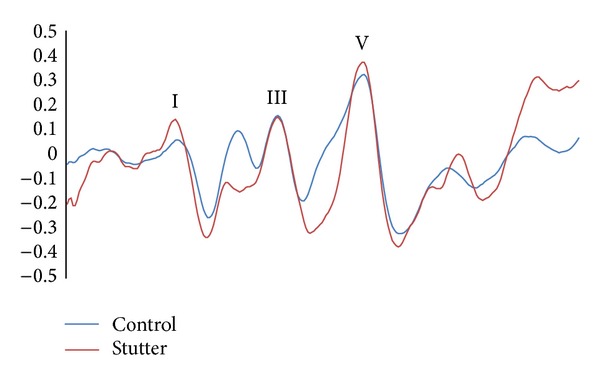
Grand average waveforms for the click evoked ABR in stutterers and controls. ABR waves I-V latency interval in stuttering adults was equivalent to normal control subjects.

Grand average waveforms for the click—ABR are shown in Figure 2. According to normative data for the click-ABR and in line with normative data in our lab the absolute latencies (I-III-V) and the interpeak latencies (I-III, III-V, and I-V) for all groups were within normal limits.  Figure 1 displays grand average responses for the speech evoked ABR in the stutterers and in the control group. As shown in Figure 1, stutterers appear to have longer latency values and shallower VA slope than the control group for the transient portion of the speech-ABR. Statistical analysis revealed significant group differences for the wave V (*t*(48) = 3.43, *P* = 0.002), wave A (*t*(48) = 2.83, *P* = 0.008), and wave O (*t*(46) = 3.66, *P* = 0.001). Based on independent samples* t*-tests stutterers had longer latencies for the onset and offset peaks relative to controls.* t*-tests comparisons yielded no significant latency differences for peak D (*t*(43) = 1.23, *P* = 0.22), peak E (*t*(46) = 1.63, *P* = 0.11), and peak F (*t*(47) = 0.59, *P* = 0.55).  Table 1 represents the mean and the standard deviation for each measure.

Analysis of composite measures (VA duration, VA inter-peak amplitude, and VA slope) revealed significant group differences for the VA slope and the stuttering group had a shallower slope compared to the control group (*t*(48) = 2.42, *P* = 0.02).

For measuring the spectral amplitude of the fundamental frequency and its harmonics (F1 & HF) Fourier analysis was used. Based on fast Fourier analysis of the speech-ABR (11.4–40.6 ms) the spectral amplitude of F0 (80–121 Hz)-F1 (454–719 Hz) and HF (721–1155 Hz) did not differ statistically between the two groups {F0 (*t*(48) = 0.060, *P* = 0.95), F1 (*t*(48) = −1.84, *P* = 0.07), and HF (*t*(48) = −0.956, *P* = 0.34)}.

To assess whether the brainstem timing deficit is related to the severity of stuttering, the latency of each peak was correlated with the score from the SSI by means of the Pearson correlation coefficient. There were significant correlations between stuttering severity and the latency of wave A (*r* = 0.45, *P* = 0.02) as well as stuttering severity and the latency of wave O (*r* = 0.84, *P* = 0.000).

## 4. Discussion

To our knowledge this is the first study of speech-ABR in persistent developmental stuttering. In this study we investigated brainstem neural synchrony via the speech evoked ABR in a group of adults with persistent developmental stuttering. ABR components (I-V, I-III, and III-V) were equivalent to normative data for all subjects. The main findings of our investigation in the speech-ABR were significantly increased waves V, A, and O latencies, as well as a shallower VA slope in the stuttering group relative to the control group. We also found significant correlations between the latencies of transient measures (wave A and wave O) and stuttering severity.

In agreement with Kent's (1983) hypothesis the results presented in our study demonstrate that the brainstem response to transient events is less synchronous in PDS subjects as compared to controls.

### 4.1. Click Evoked ABR

According to normative data for the click-ABR and in line with normative data in our lab absolute latencies (I-III-V) and interpeak latencies (I-III, III-V, and I-V) for all groups (stutterers and control group) were within normal limits. The results regarding theauditory brainstem responses in stuttering are, however, contradictory. Some researchers found significant differences in ABR components between stutterers and nonstutterers [19, 20]. In contrast and in line with our results, others questioned these findings and failed to show differences in central conduction time of ABRs between stutterers and controls [28, 29]. Some of the disparate findings in this regard are due to differences in the methodology of studies and pathological heterogeneity of stuttering.

Interestingly, this study showed that at least in a subgroup of stutterers, alterations in neural synchrony in the brainstem response to/da/stimuli may occur prior to any significant shifts in the latencies of the click-ABR components. This result indicates that an ABR with normal latency values does not exclude the possibility of timing deficits in the midbrain. Despite the high sensitivity of electrophysiological tests such as ABR in central lesion at the caudal part of the brainstem, they may fail in detecting subtle central auditory deficits at the upper levels. Masuda et al. described a report in which partial inferior colliculus destruction and medial geniculate body degeneration had no effect on the click-ABR [30]. Thus it seems that the speech-ABR gives further information about subcortical information processing complementary to that obtained by the click-ABR [31].

### 4.2. Speech Evoked ABR

Analysis of timing measures showed statistically significant difference between the two groups for the aperiodic part of the response. The onset peaks (waves A and V) were significantly longer for stutterers versus control group and the majority of subjects in stuttering group had abnormal delay for the offset peak (wave O). The presence of aberrant brainstem timing in PDS subjects demonstrates that the neural response to rapid acoustic transients is less synchronous in PDS subjects as compared to controls.

In our study, adults with PDS showed FFR interwave latency values comparable to that of the control group and comparison of the F0 magnitude between stutterers and nonstutterers yielded no significant magnitude differences. The lack of magnitude differences in the frequency encoding components (fundamental frequency, first formant, and high frequencies: frequencies between F1 and F2) shows that neither pitch nor harmonic encoding is impaired in stuttering.

In line with the results of behavioral investigations of central auditory processing in persistent stuttering, the differences between groups in the onset and offset timing (V-A-O), but not in the F1 and HF magnitudes, demonstrate that problem exists in temporal processing rather than spectral encoding. The results of the present study are consistent with Kramer et al. [16]. They found no group differences on the synthetic sentence identification-ipsilateral competing message (SSI-ICM) between stutterers and nonstutterers. The identification of the target sentence in the SSI-ICM largely relies on spectral processing. On the other hand, their findings on the binaural masking level difference demonstrated that adults with persistent developmental stuttering had significantly poorer temporal processing compared to fluent speakers.

Two likely explanations can be described for these temporal abnormalities. One explanation for the shallower slope and longer latencies of the fast onset components of speech-ABR in subjects with PDS may be linked to the timing disturbance in the auditory pathways, which results in asynchronous transmission of auditory afferent information and inefficient processing of stop consonants. Speech encoding requires precise temporal information [9]. Thus, the involvement of the neural generators of onset and/or offset responses within the brainstem might be the cause of brainstem timing deficits in stutterers.

Another explanation is top down influences. Subcortical speech encoding is affected by top down processes (memory, language experience, and attention) through the corticofugal system and such effects can change the response properties of the neurons within the brainstem structures [32]. Further evidence of an association between auditory function at the brainstem and the cortex can be seen in studies showing abnormal brainstem timing in subjects with cortical dysfunction [31]. Since there are projections from the cortex to the rostral and caudal part of the brainstem, it could be inferred that cortical dysfunction leads to aberrant corticofugal feedback on the subcortical regions, which eventually produces impaired neural synchrony at the level of the brainstem. This argument is also supported by animal models, in which ablation of primary auditory cortex changed the neural response properties in the inferior colliculus [32]. Whether asynchronous response to rapid acoustic transients can be related to bottom up mechanisms or result from the top down mechanisms is still debated. It appears reasonable that both are engaged, although the range of contribution of each is unknown.

The most likely explanation for different findings of the speech and click evoked responses is that the stimuli used were not comparable. It has been suggested that different mechanisms are likely participated to encode the click versus speech stimuli. Because of backward masking effect (effect of vowel on brief consonant), the speech stimuli may be more challenging to the central auditory system. Discrepancy between the encoding of these stimuli indicates that abnormal timing deficit as revealed by the speech-ABRs is related to differences in synchronization of response generators. The outcomes of the current investigation suggest that PDS subjects have deficient neural timing in response to the onset and offset of speech stimulus. In this study, despite the lack of group differences in the encoding of source cues (information relating F0 or pitch encoding), there were significant differences for some of filter cues (information relating timing and harmonic encoding). These results indicate that stuttering is associated with subtle impairment of speech encoding at the brainstem.

### 4.3. Clinical Implication

Cognitive factors (such as working memory and attention) are linked to speech perception and production. Considering the effects of high level cognitive processes on the speech-ABR [33, 34], we suppose that the speech-ABR can be used as an objective means for monitoring the stuttering remediation following speech fluency shaping programs in subjects who stutter. Further investigation is needed to confirm this assumption. The information gathered from this study can be used to improve our understanding of persistent developmental stuttering and its relationship to auditory abnormality. On the basis of current studies, the brainstem response to the synthesized speech sounds seems to have considerable promise for utilization in clinical populations. Nevertheless, further research is needed before it can receive widespread acceptance.

### 4.4. Conclusion

These findings demonstrated that adults with persistent developmental stuttering have neural encoding deficits for timing features at the early stages of the auditory pathway. Furthermore, these results provide a positive correlation between stuttering severity and auditory perceptual deficit in developmental stuttering which demonstrate the relevance of speech perception networks to speech production.

## Figures and Tables

**Table 1:** Mean and standard deviation for latency, composite, and spectral measures.

	Control	PDS	*P* value
Latency measures (ms)			
V	6.57 (0.09)	6.75 (0.24)	0.002
A	7.54 (0.18)	7.80 (0.41)	0.008
D	22.57 (0.44)	22.73 (0.45)	0.22
E	30.91 (0.33)	31.09 (0.41)	0.11
F	39.58 (0.46)	39.63 (0.44)	0.55
O	48.08 (0.25)	48.41 (0.36)	0.001
Composite measures			
VA duration (ms)	0.96 (0.12)	1.06 (0.24)	0.09
VA amp (*μ*V)	0.31 (0.08)	0.28 (0.10)	0.21
VA slope (ms/lV)	−0.33 (0.08)	−0.27 (0.09)	0.02
Spectral measures (*μ*V)			
F0	0.0480 (0.024)	0.0483 (0.017)	0.95
F1	0.0088 (0.003)	0.0074 (0.002)	0.07
HF	0.0036 (0.001)	0.0032 (0.001)	0.34

PDS: persistent developmental stuttering.

**Table 2:** Demographic characteristics of stutterers and controls.

	Stutterers	Controls
Number of subjects	25	25
Gender	21 M/4 F	21 M/4 F
Age	24.48 ± 3.99	22.44 ± 2.32
Education	12.2 ± 4.2	13.5 ± 1.2
